# Bis(1,10-phenanthroline-κ^2^ *N*,*N*′)(sulfato-κ^2^ *O*,*O*′)cobalt(II) propane-1,3-diol solvate

**DOI:** 10.1107/S1600536810003478

**Published:** 2010-02-03

**Authors:** Kai-Long Zhong

**Affiliations:** a Department of Applied Chemistry, Nanjing College of Chemical Technology, Nanjing 210048, People’s Republic of China

## Abstract

The title compound, [Co(SO_4_)(C_12_H_8_N_2_)_2_]·C_3_H_8_O_2_, was obtained unexpectedly as a by-product during an attempt to synthesize a mixed-ligand complex of Co^II^ with 1,10-phenanthroline (phen) and melamine *via* a solvothermal reaction. The Co^II^ metal ions are in a distorted octa­hedral coordination environment formed by four N atoms from two chelating phen ligands and two O atoms from a bidentate sulfate ligand. The two chelating N_2_C_2_ groups are almost perpendicular to each other [dihedral angle = 80.06 (8)°]. A twofold rotation axis passes through the Co and S atoms, and also through the central C atom of the propane-1,3-diol solvent mol­ecule. Inter­molecular O—H⋯O hydrogen bonds help to stabilize the structure.

## Related literature

For related cobalt compounds with monodentate, bidentate-bridging sulfate ligands, see: Hennig *et al.* (1975 ▶); Li & Zhou (1987 ▶); Song *et al.* (2008 ▶); Zheng & Lin (2003 ▶). For related complexes with bidentate-chelating ligands, see: Lu *et al.* (2006 ▶); Paul *et al.* (2002 ▶); Wang *et al.* (2009 ▶). For an isostructural structure, see: Zhong *et al.* (2006 ▶). For a related structure, see: Chen *et al.* (2005 ▶). For applications of transition metal complexes of phen, see: Li *et al.* (2004 ▶); Wang *et al.* (2000 ▶).
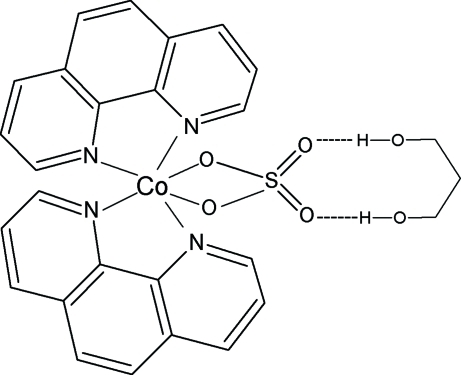


## Experimental

### 

#### Crystal data

[Co(SO_4_)(C_12_H_8_N_2_)_2_]·C_3_H_8_O_2_
*M* *_r_* = 591.49
Monoclinic, 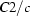

*a* = 18.285 (4) Å
*b* = 12.422 (3) Å
*c* = 13.211 (3) Å
β = 121.82 (3)°
*V* = 2549.7 (13) Å^3^
*Z* = 4
Mo *K*α radiation
μ = 0.81 mm^−1^
*T* = 223 K
0.60 × 0.40 × 0.34 mm

#### Data collection

Rigaku Mercury CCD diffractometer
Absorption correction: multi-scan (*REQAB*: Jacobson, 1998 ▶) *T*_min_ = 0.823, *T*_max_ = 1.000
7099 measured reflections
2898 independent reflections
2540 reflections with *I* > 2σ(*I*)
*R*_int_ = 0.021

#### Refinement

*R*[*F* ^2^ > 2σ(*F* ^2^)] = 0.038
*wR*(*F* ^2^) = 0.101
*S* = 1.06
2898 reflections
183 parameters
21 restraints
H atoms treated by a mixture of independent and constrained refinement
Δρ_max_ = 0.72 e Å^−3^
Δρ_min_ = −0.45 e Å^−3^

### 

Data collection: *CrystalClear* (Rigaku, 2007 ▶); cell refinement: *CrystalClear*; data reduction: *CrystalClear*; program(s) used to solve structure: *SHELXS97* (Sheldrick, 2008 ▶); program(s) used to refine structure: *SHELXL97* (Sheldrick, 2008 ▶); molecular graphics: *XP* in *SHELXTL* (Sheldrick, 2008 ▶); software used to prepare material for publication: *SHELXTL*.

## Supplementary Material

Crystal structure: contains datablocks global, I. DOI: 10.1107/S1600536810003478/zq2024sup1.cif

Structure factors: contains datablocks I. DOI: 10.1107/S1600536810003478/zq2024Isup2.hkl

Additional supplementary materials:  crystallographic information; 3D view; checkCIF report

## Figures and Tables

**Table 1:** Hydrogen-bond geometry (Å, °)

*D*—H⋯*A*	*D*—H	H⋯*A*	*D*⋯*A*	*D*—H⋯*A*
O3—H3*B*⋯O2	0.82	1.92	2.737 (3)	179

**Fig. 1.**
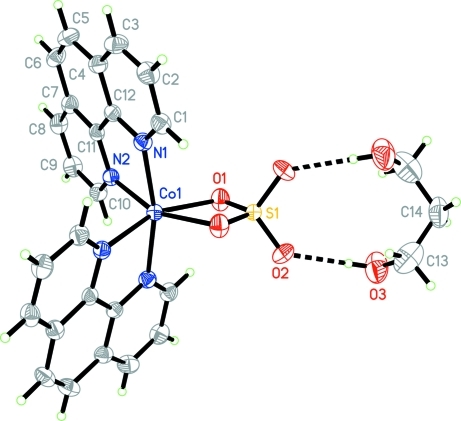
The molecular structure of the title complex showing the atom-numbering scheme and with displacement ellipsoids drawn at the 50% probability level. The dashed lines represent O—H···O interactions. Unlabeled atoms are related to the labelled atoms by the symmetry operator (-x + 1, y, - z + 1/2).

## supplementary crystallographic information

### Comment

Metal-organic complexes have been widely applied in fields of research and production because of their capability of showing novel optical, electronical, magnetical properties. M-phen transition metal complexes (phen = phenantroline) possess important actions in the areas of extraction, plating, bio-inorganic chemistry, analytical chemistry and functional materials (Wang *et al.*, 2000; Li *et al.*, 2004).

Although many transtion metal complexes with sulfate ions as monodentate, bidentate and bidentate-bridging ligands have been structurally characterized (Hennig *et al.*, 1975; Zheng & Lin, 2003; Song *et al.*, 2008), as far as we know, the reports on complexes with bidentate-chelating sulfate ligands are few (Paul *et al.*, 2002; Lu *et al.*, 2006; Wang *et al.*, 2009). Here, we report the crystal structure of the new complex [CoSO_4_(C_12_H_8_N_2_)_2_].C_3_H_8_O_2 _unexpectedly obtained during an attempt to synthesize a mixed-ligand complex of cobalt with phen and melamine *via* a solvothermal reaction, which is analogue with a previously reported Co^II^ complex (Zhong *et al.*, 2006).

In the crystal structure of the title complex, the Co^II^ metal ion is six-coordinated in a distorted octahedral environement by four N atoms from two chelating phen ligands and two O atoms from a bidentate-chelating sulfate ligand (Fig. 1). The Co—O bond distance [2.1323 (15) Å], the O—Co—O bite angle [66.54 (8)°], the Co—N bond distances [2.1295 (16)–2.1341 (17) Å] and the N—Co—N bite angle [77.99 (6)°] are very similar to those seen in the previously reported cobalt complex [CoSO_4_(C_12_H_8_N_2_)_2_].C_2_H_6_O_2 _(II)[Zhong *et al.*, 2006]. The dihedral angle between the two chelating N_2_C_2_ groups is 80.06 (8)°, this is larger than that found in (II) [70.16 (6)°]. A twofold rotation axis (symmetry code: -*x* + 1, *y*, - *z* + 1/2) passes through the Co and S atoms, and also through the mid-carbon of the propane-1,3-diol solvent molecule. The crystal structure is further stabilized by intermolecular O3—H3B···O2 hydrogen bonds (Fig. 1 and Table 1).

### Experimental

Red prism-shaped crystals of the title compound was unexpectedly obtained as a by-product during an attempt to synthesize a mixed-ligand cobalt complex with phen and melamine *via* a propane-1,3-diol/water solvothermal reaction. 0.2 mmol phen, 0.1 mmol melamine, 0.1 mmol CoSO_4_.7H_2_O, 2.0 ml 1,3-propanediol and 1.0 ml water were mixed and placed in a thick Pyrex tube, which was sealed and heated to 413 K for 96 h, whereupon red-prisms of the title complex were obtained.

### Refinement

The non-H atoms were refined anisotropically. The H atoms of phen were positioned geometrically and allowed to ride on their parent atoms, with C—H = 0.93 Å and *U*_iso_(H) = 1.2*U*_eq_(C). The H atoms of central C atom of propane-1,3-diol were constrained, with C—H = 0.97 Å and *U*_iso_(H) = 1.2*U*_eq_(C), whereas other H atoms were placed in geometrically idealized positions and refined as riding atoms, with C—H = 0.97 Å and O—H = 0.82 Å, and with *U*_iso_(H) = 1.2*U*_eq_(C) and 1.5*U*_eq_(O).

### Crystal data

**Table:**

[Co(SO_4_)(C_12_H_8_N_2_)_2_]·C_3_H_8_O_2_	*F*(000) = 1220
*M**_r_* = 591.49	*D*_x_ = 1.541 Mg m^−^^3^
Monoclinic, *C*2/*c*	Mo *K*α radiation, λ = 0.71073 Å
Hall symbol: -C 2yc	Cell parameters from 3592 reflections
*a* = 18.285 (4) Å	θ = 3.2–27.5°
*b* = 12.422 (3) Å	µ = 0.81 mm^−^^1^
*c* = 13.211 (3) Å	*T* = 223 K
β = 121.82 (3)°	Prism, red
*V* = 2549.7 (13) Å^3^	0.60 × 0.40 × 0.34 mm
*Z* = 4	

### Data collection

**Table:**

Rigaku Mercury CCD diffractometer	2898 independent reflections
Radiation source: fine-focus sealed tube	2540 reflections with *I* > 2σ(*I*)
Graphite Monochromator	*R*_int_ = 0.021
Detector resolution: 28.5714 pixels mm^-1^	θ_max_ = 27.5°, θ_min_ = 3.2°
ω scans	*h* = −23→23
Absorption correction: multi-scan (*REQAB*: Jacobson, 1998)	*k* = −16→13
*T*_min_ = 0.823, *T*_max_ = 1.000	*l* = −17→14
7099 measured reflections	

### Refinement

**Table:**

Refinement on *F*^2^	Secondary atom site location: difference Fourier map
Least-squares matrix: full	Hydrogen site location: inferred from neighbouring sites
*R*[*F*^2^ > 2σ(*F*^2^)] = 0.038	H atoms treated by a mixture of independent and constrained refinement
*wR*(*F*^2^) = 0.101	*w* = 1/[σ^2^(*F*_o_^2^) + (0.0616*P*)^2^ + 1.0504*P*] where *P* = (*F*_o_^2^ + 2*F*_c_^2^)/3
*S* = 1.06	(Δ/σ)_max_ < 0.001
2898 reflections	Δρ_max_ = 0.72 e Å^−^^3^
183 parameters	Δρ_min_ = −0.45 e Å^−^^3^
21 restraints	Extinction correction: *SHELXL97* (Sheldrick, 2008), Fc^*^=kFc[1+0.001xFc^2^λ^3^/sin(2θ)]^-1/4^
Primary atom site location: structure-invariant direct methods	Extinction coefficient: 0.0057 (6)

### Special details

**Table:**

Geometry. All e.s.d.'s (except the e.s.d. in the dihedral angle between two l.s. planes) are estimated using the full covariance matrix. The cell e.s.d.'s are taken into account individually in the estimation of e.s.d.'s in distances, angles and torsion angles; correlations between e.s.d.'s in cell parameters are only used when they are defined by crystal symmetry. An approximate (isotropic) treatment of cell e.s.d.'s is used for estimating e.s.d.'s involving l.s. planes.
Refinement. Refinement of *F*^2^ against ALL reflections. The weighted *R*-factor *wR* and goodness of fit *S* are based on *F*^2^, conventional *R*-factors *R* are based on *F*, with *F* set to zero for negative *F*^2^. The threshold expression of *F*^2^ > σ(*F*^2^) is used only for calculating *R*-factors(gt) *etc*. and is not relevant to the choice of reflections for refinement. *R*-factors based on *F*^2^ are statistically about twice as large as those based on *F*, and *R*- factors based on ALL data will be even larger.

### Fractional atomic coordinates and isotropic or equivalent isotropic displacement parameters (Å^2^)

**Table:**

	*x*	*y*	*z*	*U*_iso_*/*U*_eq_	
Co1	0.5000	0.81835 (3)	0.2500	0.02201 (14)	
S1	0.5000	1.03618 (5)	0.2500	0.02331 (17)	
O1	0.44793 (9)	0.96187 (12)	0.14805 (12)	0.0329 (3)	
C11	0.34382 (12)	0.68292 (14)	0.14757 (16)	0.0224 (4)	
N2	0.40420 (10)	0.71189 (13)	0.12195 (14)	0.0238 (3)	
C10	0.39507 (13)	0.67410 (16)	0.02169 (18)	0.0284 (4)	
H10A	0.4356	0.6935	0.0028	0.034*	
C8	0.26687 (13)	0.57717 (16)	−0.03144 (17)	0.0304 (4)	
H8A	0.2213	0.5328	−0.0832	0.036*	
O2	0.55612 (10)	1.10269 (14)	0.22920 (16)	0.0446 (4)	
C6	0.21399 (13)	0.58852 (17)	0.10868 (18)	0.0310 (4)	
H6A	0.1679	0.5434	0.0604	0.037*	
C12	0.35267 (11)	0.72718 (15)	0.25388 (16)	0.0223 (4)	
C7	0.27424 (12)	0.61511 (16)	0.07446 (17)	0.0264 (4)	
C4	0.29234 (12)	0.69936 (16)	0.28497 (17)	0.0270 (4)	
C9	0.32726 (14)	0.60646 (18)	−0.05671 (18)	0.0331 (4)	
H9A	0.3235	0.5817	−0.1257	0.040*	
C5	0.22322 (12)	0.62810 (18)	0.21042 (18)	0.0322 (5)	
H5A	0.1841	0.6086	0.2319	0.039*	
N1	0.41806 (10)	0.79727 (13)	0.31789 (14)	0.0250 (3)	
C1	0.42581 (13)	0.84107 (17)	0.41526 (18)	0.0291 (4)	
H1A	0.4706	0.8892	0.4597	0.035*	
C2	0.36899 (14)	0.81726 (18)	0.45310 (19)	0.0335 (5)	
H2A	0.3762	0.8491	0.5215	0.040*	
C3	0.30319 (13)	0.74709 (18)	0.38888 (18)	0.0335 (5)	
H3A	0.2653	0.7306	0.4135	0.040*	
O3	0.56334 (16)	1.31539 (16)	0.1809 (2)	0.0659 (6)	
H3B	0.5610	1.2514	0.1944	0.099*	
C14	0.5000	1.4456 (3)	0.2500	0.0562 (10)	
C13	0.5774 (2)	1.3764 (3)	0.2780 (3)	0.0723 (9)	
H13A	0.6268	1.4227	0.3033	0.087*	
H13B	0.5907	1.3286	0.3436	0.087*	
H14	0.496 (3)	1.442 (4)	0.174 (2)	0.143 (19)*	

### Atomic displacement parameters (Å^2^)

**Table:**

	*U*^11^	*U*^22^	*U*^33^	*U*^12^	*U*^13^	*U*^23^
Co1	0.0211 (2)	0.0208 (2)	0.0239 (2)	0.000	0.01172 (16)	0.000
S1	0.0212 (3)	0.0200 (3)	0.0303 (3)	0.000	0.0146 (3)	0.000
O1	0.0315 (7)	0.0277 (8)	0.0270 (7)	0.0012 (6)	0.0068 (6)	0.0011 (6)
C11	0.0222 (9)	0.0193 (9)	0.0227 (9)	0.0027 (7)	0.0098 (7)	0.0026 (7)
N2	0.0253 (8)	0.0218 (8)	0.0254 (8)	0.0006 (6)	0.0141 (7)	0.0004 (6)
C10	0.0334 (10)	0.0272 (11)	0.0284 (10)	−0.0004 (8)	0.0189 (9)	−0.0014 (8)
C8	0.0306 (10)	0.0278 (11)	0.0241 (9)	−0.0033 (8)	0.0085 (8)	−0.0045 (8)
O2	0.0435 (9)	0.0362 (9)	0.0676 (12)	−0.0093 (8)	0.0386 (9)	0.0028 (8)
C6	0.0243 (9)	0.0314 (11)	0.0298 (10)	−0.0075 (8)	0.0092 (8)	0.0014 (8)
C12	0.0202 (8)	0.0222 (9)	0.0218 (8)	0.0026 (7)	0.0092 (7)	0.0028 (7)
C7	0.0251 (9)	0.0245 (10)	0.0247 (9)	0.0007 (8)	0.0098 (8)	0.0020 (7)
C4	0.0251 (9)	0.0291 (10)	0.0271 (9)	0.0023 (8)	0.0139 (8)	0.0045 (8)
C9	0.0397 (11)	0.0332 (11)	0.0252 (9)	0.0002 (9)	0.0162 (9)	−0.0043 (8)
C5	0.0259 (9)	0.0382 (12)	0.0328 (10)	−0.0041 (9)	0.0157 (8)	0.0046 (9)
N1	0.0249 (8)	0.0252 (8)	0.0253 (8)	0.0004 (6)	0.0135 (7)	−0.0008 (6)
C1	0.0304 (10)	0.0283 (11)	0.0270 (9)	−0.0003 (8)	0.0140 (8)	−0.0050 (8)
C2	0.0409 (12)	0.0361 (12)	0.0282 (10)	0.0029 (9)	0.0213 (10)	−0.0025 (8)
C3	0.0347 (10)	0.0419 (13)	0.0318 (10)	0.0004 (9)	0.0229 (9)	0.0030 (9)
O3	0.1116 (18)	0.0412 (11)	0.0867 (15)	−0.0012 (11)	0.0808 (15)	0.0034 (10)
C14	0.085 (3)	0.0301 (18)	0.068 (3)	0.000	0.050 (2)	0.000
C13	0.077 (2)	0.072 (2)	0.069 (2)	−0.0252 (18)	0.0392 (17)	−0.0071 (17)

### Geometric parameters (Å, °)

**Table:**

Co1—N1^i^	2.1295 (16)	C6—C7	1.432 (3)
Co1—N1	2.1295 (16)	C6—H6A	0.9300
Co1—O1^i^	2.1323 (15)	C12—N1	1.355 (3)
Co1—O1	2.1323 (15)	C12—C4	1.408 (3)
Co1—N2	2.1341 (17)	C4—C3	1.410 (3)
Co1—N2^i^	2.1341 (17)	C4—C5	1.428 (3)
Co1—S1	2.7058 (9)	C9—H9A	0.9300
S1—O2	1.4519 (15)	C5—H5A	0.9300
S1—O2^i^	1.4519 (15)	N1—C1	1.334 (3)
S1—O1	1.4901 (15)	C1—C2	1.401 (3)
S1—O1^i^	1.4901 (15)	C1—H1A	0.9300
C11—N2	1.363 (2)	C2—C3	1.360 (3)
C11—C7	1.402 (3)	C2—H2A	0.9300
C11—C12	1.436 (3)	C3—H3A	0.9300
N2—C10	1.330 (2)	O3—C13	1.392 (3)
C10—C9	1.401 (3)	O3—H3B	0.8200
C10—H10A	0.9300	C14—C13^i^	1.525 (4)
C8—C9	1.361 (3)	C14—C13	1.525 (4)
C8—C7	1.414 (3)	C14—H14	0.97 (3)
C8—H8A	0.9300	C13—H13A	0.9700
C6—C5	1.356 (3)	C13—H13B	0.9700
			
N1^i^—Co1—N1	165.87 (9)	C9—C8—H8A	120.4
N1^i^—Co1—O1^i^	100.94 (6)	C7—C8—H8A	120.4
N1—Co1—O1^i^	90.91 (6)	C5—C6—C7	120.98 (18)
N1^i^—Co1—O1	90.91 (6)	C5—C6—H6A	119.5
N1—Co1—O1	100.94 (6)	C7—C6—H6A	119.5
O1^i^—Co1—O1	66.54 (8)	N1—C12—C4	123.01 (17)
N1^i^—Co1—N2	93.19 (6)	N1—C12—C11	117.63 (16)
N1—Co1—N2	77.99 (6)	C4—C12—C11	119.33 (17)
O1^i^—Co1—N2	157.72 (6)	C11—C7—C8	117.41 (18)
O1—Co1—N2	96.36 (6)	C11—C7—C6	119.39 (18)
N1^i^—Co1—N2^i^	77.99 (6)	C8—C7—C6	123.19 (18)
N1—Co1—N2^i^	93.19 (6)	C12—C4—C3	116.81 (18)
O1^i^—Co1—N2^i^	96.36 (6)	C12—C4—C5	119.71 (18)
O1—Co1—N2^i^	157.72 (6)	C3—C4—C5	123.45 (18)
N2—Co1—N2^i^	103.42 (9)	C8—C9—C10	119.69 (19)
N1^i^—Co1—S1	97.06 (5)	C8—C9—H9A	120.2
N1—Co1—S1	97.06 (5)	C10—C9—H9A	120.2
O1^i^—Co1—S1	33.27 (4)	C6—C5—C4	120.73 (18)
O1—Co1—S1	33.27 (4)	C6—C5—H5A	119.6
N2—Co1—S1	128.29 (5)	C4—C5—H5A	119.6
N2^i^—Co1—S1	128.29 (5)	C1—N1—C12	118.30 (16)
O2—S1—O2^i^	110.64 (15)	C1—N1—Co1	127.99 (14)
O2—S1—O1	111.10 (9)	C12—N1—Co1	113.67 (12)
O2^i^—S1—O1	110.18 (9)	N1—C1—C2	122.42 (19)
O2—S1—O1^i^	110.18 (9)	N1—C1—H1A	118.8
O2^i^—S1—O1^i^	111.10 (9)	C2—C1—H1A	118.8
O1—S1—O1^i^	103.45 (12)	C3—C2—C1	119.41 (19)
O2—S1—Co1	124.68 (7)	C3—C2—H2A	120.3
O2^i^—S1—Co1	124.68 (7)	C1—C2—H2A	120.3
O1—S1—Co1	51.72 (6)	C2—C3—C4	120.05 (18)
O1^i^—S1—Co1	51.72 (6)	C2—C3—H3A	120.0
S1—O1—Co1	95.01 (7)	C4—C3—H3A	120.0
N2—C11—C7	123.19 (17)	C13—O3—H3B	109.5
N2—C11—C12	116.97 (16)	C13^i^—C14—C13	111.4 (3)
C7—C11—C12	119.82 (17)	C13^i^—C14—H14	100 (3)
C10—N2—C11	117.47 (17)	C13—C14—H14	77 (3)
C10—N2—Co1	128.88 (13)	O3—C13—C14	112.8 (2)
C11—N2—Co1	113.62 (12)	O3—C13—H13A	109.0
N2—C10—C9	123.06 (18)	C14—C13—H13A	109.0
N2—C10—H10A	118.5	O3—C13—H13B	109.0
C9—C10—H10A	118.5	C14—C13—H13B	109.0
C9—C8—C7	119.17 (19)	H13A—C13—H13B	107.8
			
N1^i^—Co1—S1—O2	−9.86 (10)	C11—N2—C10—C9	−0.4 (3)
N1—Co1—S1—O2	170.14 (10)	Co1—N2—C10—C9	−178.16 (15)
O1^i^—Co1—S1—O2	89.33 (12)	N2—C11—C12—N1	2.5 (3)
O1—Co1—S1—O2	−90.67 (12)	C7—C11—C12—N1	−176.19 (17)
N2—Co1—S1—O2	−109.61 (10)	N2—C11—C12—C4	−179.60 (17)
N2^i^—Co1—S1—O2	70.39 (10)	C7—C11—C12—C4	1.7 (3)
N1^i^—Co1—S1—O2^i^	170.14 (10)	N2—C11—C7—C8	−1.0 (3)
N1—Co1—S1—O2^i^	−9.86 (10)	C12—C11—C7—C8	177.62 (17)
O1^i^—Co1—S1—O2^i^	−90.67 (12)	N2—C11—C7—C6	179.73 (18)
O1—Co1—S1—O2^i^	89.33 (12)	C12—C11—C7—C6	−1.7 (3)
N2—Co1—S1—O2^i^	70.39 (10)	C9—C8—C7—C11	0.8 (3)
N2^i^—Co1—S1—O2^i^	−109.61 (10)	C9—C8—C7—C6	−179.9 (2)
N1^i^—Co1—S1—O1	80.81 (9)	C5—C6—C7—C11	0.1 (3)
N1—Co1—S1—O1	−99.19 (9)	C5—C6—C7—C8	−179.1 (2)
O1^i^—Co1—S1—O1	180.0	N1—C12—C4—C3	−0.9 (3)
N2—Co1—S1—O1	−18.94 (9)	C11—C12—C4—C3	−178.66 (17)
N2^i^—Co1—S1—O1	161.06 (9)	N1—C12—C4—C5	177.55 (18)
N1^i^—Co1—S1—O1^i^	−99.19 (9)	C11—C12—C4—C5	−0.2 (3)
N1—Co1—S1—O1^i^	80.81 (9)	C7—C8—C9—C10	−0.5 (3)
O1—Co1—S1—O1^i^	180.0	N2—C10—C9—C8	0.3 (3)
N2—Co1—S1—O1^i^	161.06 (9)	C7—C6—C5—C4	1.4 (3)
N2^i^—Co1—S1—O1^i^	−18.94 (9)	C12—C4—C5—C6	−1.3 (3)
O2—S1—O1—Co1	118.19 (9)	C3—C4—C5—C6	177.0 (2)
O2^i^—S1—O1—Co1	−118.83 (9)	C4—C12—N1—C1	0.6 (3)
O1^i^—S1—O1—Co1	0.0	C11—C12—N1—C1	178.38 (17)
N1^i^—Co1—O1—S1	−101.54 (8)	C4—C12—N1—Co1	178.28 (14)
N1—Co1—O1—S1	86.20 (8)	C11—C12—N1—Co1	−3.9 (2)
O1^i^—Co1—O1—S1	0.0	N1^i^—Co1—N1—C1	128.19 (17)
N2—Co1—O1—S1	165.15 (7)	O1^i^—Co1—N1—C1	−19.01 (17)
N2^i^—Co1—O1—S1	−42.22 (19)	O1—Co1—N1—C1	−85.28 (18)
C7—C11—N2—C10	0.7 (3)	N2—Co1—N1—C1	−179.54 (18)
C12—C11—N2—C10	−177.90 (17)	N2^i^—Co1—N1—C1	77.41 (18)
C7—C11—N2—Co1	178.87 (14)	S1—Co1—N1—C1	−51.81 (17)
C12—C11—N2—Co1	0.2 (2)	N1^i^—Co1—N1—C12	−49.27 (13)
N1^i^—Co1—N2—C10	−14.97 (17)	O1^i^—Co1—N1—C12	163.52 (13)
N1—Co1—N2—C10	176.18 (18)	O1—Co1—N1—C12	97.25 (14)
O1^i^—Co1—N2—C10	114.64 (19)	N2—Co1—N1—C12	2.99 (13)
O1—Co1—N2—C10	76.30 (17)	N2^i^—Co1—N1—C12	−100.06 (14)
N2^i^—Co1—N2—C10	−93.38 (17)	S1—Co1—N1—C12	130.73 (13)
S1—Co1—N2—C10	86.62 (17)	C12—N1—C1—C2	−0.1 (3)
N1^i^—Co1—N2—C11	167.15 (13)	Co1—N1—C1—C2	−177.43 (15)
N1—Co1—N2—C11	−1.71 (12)	N1—C1—C2—C3	−0.1 (3)
O1^i^—Co1—N2—C11	−63.2 (2)	C1—C2—C3—C4	−0.3 (3)
O1—Co1—N2—C11	−101.58 (13)	C12—C4—C3—C2	0.7 (3)
N2^i^—Co1—N2—C11	88.74 (13)	C5—C4—C3—C2	−177.6 (2)
S1—Co1—N2—C11	−91.26 (13)	C13^i^—C14—C13—O3	64.6 (2)

Symmetry codes: (i) −*x*+1, *y*, −*z*+1/2.

### Hydrogen-bond geometry (Å, °)

**Table:**

*D*—H···*A*	*D*—H	H···*A*	*D*···*A*	*D*—H···*A*
O3—H3B···O2	0.82	1.92	2.737 (3)	179
